# Induction of humoral immune responses and inhibition of metastasis in mice by a VEGF peptide-based vaccine

**DOI:** 10.22038/ijbms.2020.38508.9141

**Published:** 2020-04

**Authors:** Faezeh Soltanpour Gharibdousti, Banafsheh Fazeli Delshad, Reza Falak, Nasrin Shayanfar, Mazdak Ganjalikhani Hakemi, Alireza Andalib, Gholam Ali Kardar

**Affiliations:** 1 Department of Immunology, School of Medicine, Isfahan University of Medical Sciences, Isfahan, Iran; 2 Immunology Research Center, School of Medicine, Iran University of Medical Sciences, Tehran, Iran; 3 Department of Pathology, School of Medicine, Iran University of Medical Sciences, Tehran, Iran; 4 Immunology, Asthma and Allergy Research Institute, Tehran University of Medical Sciences, Tehran, Iran

**Keywords:** Angiogenesis, Immunoinformatics, Metastasis, Peptide-based vaccine, VEGF

## Abstract

**Objective(s):**

Blocking of vascular endothelial growth factor (VEGF) plays a pivotal role in inhibition of metastasis and is a target for development of anti-angiogenic agents. In this study, a peptide-based vaccine was designed and its potential for induction of humoral immune responses, generation of neutralizing antibodies, inhibition of tumor growth and metastasis was determined.

**Materials and Methods:**

With online bioinformatics tools, a fragment of the VEGF-A was selected for a peptide-based vaccine. To enhance its antigenicity, the peptide was conjugated with *Keyhole limpet hemocyanin* and used to immunize mice. Then, the polyclonal anti-VEGF antibody titer was measured and its effect on proliferation of HUVEC cell line was investigated by MTT assay. Finally, we checked the effect of the peptide on tumor growth, metastasis, and survival rates in a mouse model of cancer.

**Results:**

The bioinformatics analysis of the selected region confirmed dis-similarity of the peptide with any other human protein and its acceptable antigenicity to stimulate a tumor-specific humoral response. Anti-VEGF antibody titers were significantly greater in vaccinated mice than in controls. IgG antibody from mice immunized with recombinant VEGF-A inhibited HUVEC proliferation (*P<*0.0001). Tumors in vaccinated mice were significantly smaller than those in controls. Moreover, metastasis was reduced and survival rates increased in the vaccinated group.

**Conclusion:**

Production of high-titer antibody against the peptide vaccine indicated that the peptide has the potency to be used as a peptide-based vaccine for humoral inhibition of tumor growth and metastasis. The efficacy of the peptide should be further tested in primate models.

## Introduction

Angiogenesis is an essential process in the development and growth of tumor tissue. In the absence of this process, tumors will not grow beyond two mm^3^ in size. Thus, inhibition of angiogenesis is a basic strategy in cancer vaccination approaches (1, 2). Sprouting of angiogenesis is regulated by various key agents such as vascular endothelial growth factor (VEGF), which is highly expressed and continuously secreted from most solid tumors, and demonstrates both autocrine and endocrine characteristics (3-5). Therefore, inhibition of angiogenesis via blockage of VEGF signaling could suppress promotion of solid tumors (6). Currently, several anti-angiogenic approaches, especially anti-VEGF drugs have been approved by international committees for certain advanced cancers; some have been applied in clinical trials, while others are under further evaluation or await approval (2). It has been previously shown that the functionality of VEGF/VEGFR could be directly blocked by various antibodies such as Bevacizumab (Avastin^®^) and Ramucirumab (Cyramza^®^), or trapped by soluble VEGF receptors such as Aflibercept (Zaltrap^®^) (7, 8), or indirectly blocked by inhibiting receptor activity by specific antibodies or administration of the inhibitors of tyrosine kinase receptor. In this regard, clinical application of Sunitinib (Sutent^®^), Sorafenib (Nexavar^®^), and Pazopanib (Votrient^®^) has been approved for various tumors (9). Bevacizumab, the first humanized monoclonal antibody against VEGF-A, was approved by the FDA in 2004. Administration of Bevacizumab, in combination with other chemotherapy agents, showed acceptable anti-tumor efficacy for many solid tumors (10). 

Among the recent approaches in cancer immunotherapy, passive immunotherapy including application of blocking monoclonal antibodies or trapping soluble receptors, merely provides a short-lived response, requiring repeated administration of the component; however, active immunotherapy that engages the host immune response is highly desirable, resulting in sustained induction of anti-cancer effects (11, 12). Anti-angiogenic vaccines could be produced based on DNA, peptide, or protein-based structures. Recently, studies focused on VEGF and its receptors as desirable targets to block angiogenesis.

Activation of patients own immune responses would likely introduce less toxicity and side effects than conventional chemotherapeutic medications. Therefore, it seems that application of protein- or peptide-based vaccines, in combination with mild doses of radiotherapy, chemotherapy, or other anti-angiogenic agents, may suppress tumor size, growth rate, and metastasis (12-16). 

In the present study, we investigated the anti-angiogenic efficacy of a VEGF-A peptide-based vaccine in mice. 

## Materials and Methods

***Immunoinformatics analysis and peptide design***

Amino acid sequences of human VEGF-A (hVEGF_165_ and hVEGF_121_) and mouse VEGF-A (mVEGF_164_ and mVEGF_120_) were retrieved from the UniProt database. Among all isoforms of human VEGF-A, human VEGF_165_ (P15692-4) and VEGF_121_ (P15692-9) were used for peptide design, because they were regarded as major isoforms of human VEGF-A, and showed no significant differences with the sequences of mouse VEGF-A_164_ (Q00731-2) and VEGF-A_120 _(Q00731-3) (2). Mega-4 alignment software was used to align the isoforms and to find the conserved and similar peptide sequences of human and mice. Among the protected areas, an extracellular 41-mer peptide was selected for determination of T- and B-cell epitopes by Immune Epitope Database Analysis Resources (IEDB). The selected areas were also evaluated for immunogenicity, hydrophilicity, antigenicity, flexibility, and accessibility. Presuming the possible future application of this peptide in human vaccination studies, during sequence selection steps, we considered the major histocompatability complex (MHC) restriction concept, possible loading of this peptide onto common human leukocyte antigen (HLA) class I and II molecules, especially in the Iranian population with HLA-A*0201 and HLA-DRB1* 1103/1104, respectively and its presentation to T cells. Non-overlapping characteristics of the selected sequence with human proteins were determined by protein BLAST. The peptide was then synthesized and attached to Keyhole limpet hemocyanin (KLH) at Selleckchem Company (Selleckchem, USA). 

***Animals***

Twenty 6-8-week-old female BALB/c mice were obtained from the animal breeding laboratory of Iran University of Medical Sciences (IUMS), Tehran, Iran, and kept for one week at the animal house to become adapted to study conditions. All procedures were performed under the rules and guidance of the Animal Care Ethical Committee at Isfahan University of Medical Sciences. Mice were divided to four groups of five mice each. The groups were: (1) the vaccine group, which was vaccinated with the KLH-conjugated VEGF-A peptide and challenged with breast cancer-inducing 4T1 cells to evaluate the prophylactic potential of the peptide, (2) the tumor group, which was injected only with 4T1 cells to evaluate the ability of the injected cells to induce tumors, (3) the antibody control group, which was vaccinated only with the KLH-conjugated peptide to evaluate antibody development, and (4) the negative control group, which received only phosphate-buffered saline (PBS).

***Immunization of mice and production of polyclonal antibody***

To induce humoral immune responses, the vaccine and antibody control group mice were injected with 100 μl of a 1 μg/μl solution of the KLH-conjugated peptide without adjuvant on days 1 of weeks 1, 3, and 5 as previously described (17). To boost the antibody titer, the antibody control group received another immunization on day 1 of week 11, too. Sera was collected from all mice before and two weeks after immunizations and stored at -20 ^°^C. 

Total IgG was isolated from the antibody control mice sera using an Antibody Purification Kit (Abcam, UK) containing protein G-coupled resin, according to the manufacturer’s instructions. The quality of the purification step was determined by SDS-PAGE and silver nitrate staining (18).

***Mouse tumor model***

To determine the prophylactic potency of the conjugated peptide, the vaccine and tumor groups were inoculated subcutaneously four days after the final immunization with 5×10^5^ 4T1 breast cancer cells as previously described (19). In brief, the 4T1 mouse breast cancer cells were cultivated in RPMI media supplemented with 10% fetal bovine serum (FBS). Mice were inoculated subcutaneously into four mammary fat pads on the right side with 5×10^5^ 4T1 cells suspended in 100 μl of PBS. Body weights, clinical symptoms, appearance of fur, and tumor size were recorded every other day. Tumor sizes were calculated using the formula: length×(width)^2^×0.5 as previously described (19). 

***Measurement of serum anti-VEGF titer***

An enzyme-linked immunosorbant assay (ELISA) was used to measure the polyclonal anti-VEGF antibody titer in mice sera. First, 0.5 mg of peptide was conjugated to 2 mg of bovine serum albumin (BSA) using glutaraldehyde reagent as described (20, 21). After dialysis, the final product was analyzed by SDS-PAGE and silver nitrate staining (17-22). For ELISA development, microtiter plates (Maxisorb, Costar, USA) were coated with 100 µl (2 μg/well) of BSA-conjugated peptide in 0.1 M sodium bicarbonate buffer (pH 9.6) overnight at 4 ^°^C. After washing the wells three times with PBS containing 0.05% Tween 20 (PBS-T), the plates were blocked with 2% casein for 2 hr. The plates were then washed twice and 100 μl of serially-diluted serum samples were added and incubated for 2 hr. The wells were then washed five times, and 100 μl of horseradish peroxidase-conjugated sheep anti-mouse IgG (Sigma, USA) were added to each well and incubated for 1 hr. The wells were then washed five times, and 100 μl of TMB (3,3′,5,5′-Tetramethylbenzidine) substrate were added. After 15 min of incubation in the dark, the reaction was stopped with 100 μl of 2 M sulfuric acid and the absorbance was read at 450 nm against a 630 nm reference filter on an ELISA reader. All incubations were performed at 37 ^°^C, except for the coating step. 

***Evaluation of inhibitory effects of polyclonal antibodies on HUVEC***

To determine the inhibitory effects of the raised polyclonal antibodies on human VEGF receptor 2 (VEGFR2)-positive cells, human umbilical vein endothelial cells (HUVECs) were cultivated in 96-well plates (1000 cells/well) with DMEM/F12 medium containing 2% FBS and incubated at 37 ^°^C in 5% CO_2_ for 24 hr. Three types of treatments were used. The negative control cells received 0, 25, 50, or 100 ng/ml of recombinant VEGF-A (Sigma, USA). The positive control cells, in addition to VEGF-A, received 100 ng/ml of anti-VEGF monoclonal antibody (Abnova, Taiwan). The test cells, in addition to VEGF-A, received various titers of purified IgG that were isolated from immunized mice. After 48 hr, cell proliferation was evaluated by MTT assay. MTT was added at a concentration of 0.5 mg/ml to each well, and plates were incubated in 5% CO_2_ for 4 hr at 37 ^°^C. Formazan crystals were dissolved by adding dimethyl sulfoxide, and after 15 min of incubation at 37 ^°^C the absorbance was measured at 570 nm on a micro plate reader.

***Histology***

Hematoxylin and Eosin (H&E)-stained paraffin-embedded tissues were histologically examined for metastases. Three animals from each group were euthanized at the end of week 9 and brains, livers, and lungs were removed, fixed in 4% paraformaldehyde, stained, and examined. Tumor metastases were examined in three random sections and the morphological parameters, indicating metastasis severity were scored from 0-3 (19). 

***Statistical analyses***

The student’s t-test was used to analyze specific IgG titers and mice body weights. The Mann-Whitney test was used to compare the tumor sizes between the vaccine and control groups. Survival time was analyzed by the Kaplan-Meier method and the groups were compared by the log-rank test. Findings were expressed as means±standard deviations (SDs). Results were considered signiﬁcant if the *P*-value of the result was <0.05. All analyses were performed using SPSS version 20.0 (Armonk, New York, USA) and GraphPad Prism 6.0 software (La Jolla, CA, USA). 

## Results

***Immunoinformatics data analysis***

**Figure 1:**
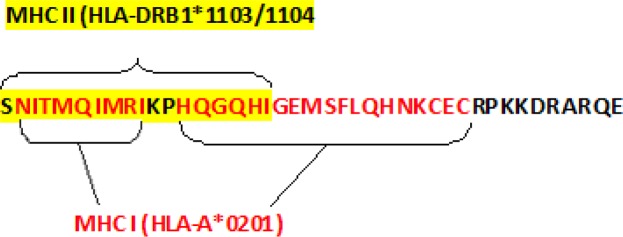
T-cell epitope prediction in human using Immune Epitope Database Analysis Resource (IEDB-AR)

The immunoinformatics study indicated that the selected 41-mer peptide (SNITMQIMRIKPHQGQHIGEMSFLQHNKCECRPKKDRARQE) was sufficiently hydrophilic and antigenic to stimulate the immune system. Based on analysis that was shown in Figure 1, selected sequence in addition to highest rank for B cell epitope has MHC I and II binding epitopes (Figure 1).

***Production and titration of anti-peptide polyclonal antibody***

**Figure 2:**
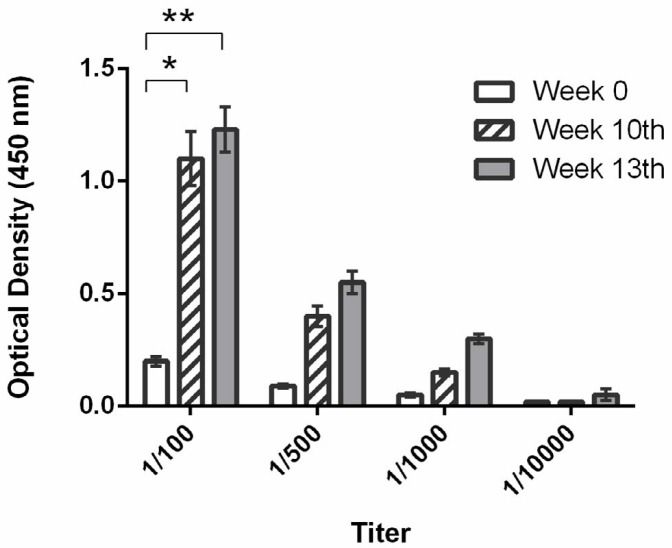
Absorbance at 450 nm of pooled sera from antibody control group mice diluted 1/100, 1/500, 1/1000, and 1/10000 on the day of immunization (day 1 of week 1) and 10 and 13 weeks after immunization (W 0, W 10, and W 13, respectively) (* *P<*0.05, ** *P<*0.005)

In the antibody control group mice, after vaccination with KLH-conjugated peptide, the absorbance at 450 nm was significantly greater at weeks 10 and 13 than on initial day of the first week in the 1/100 diluted pooled sera (*P*<0.05 and *P*<0.005, respectively), indicating that the anti-VEGF polyclonal antibody titer was significantly greater at weeks 10 and 13 than on day 1 (Figure 2). 

**Figure 3:**
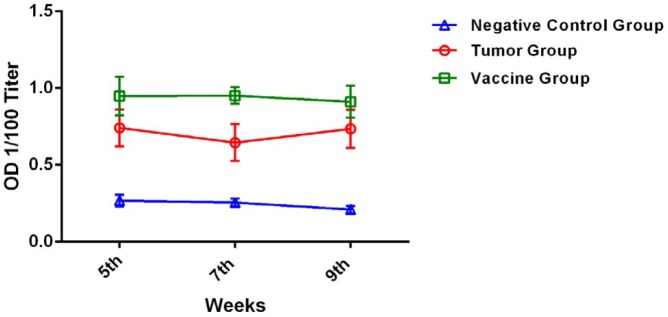
Optical densities of 1/100 dilutions of sera (means±SDs) from the tumor group and vaccine group mice, which were inoculated with 4T1 cells, and the negative control group, measured by ELISA 5, 7, and 9 weeks after immunization (*P=*0.05, *P=*0.005, respectively)

Sera were collected from the mice at 5, 7, and 9 weeks after inoculation of the tumor cells and peptide vaccine. Optical densities (ODs) of 1/100 dilutions of these sera were analyzed (Figure 3). At all three time points, the ODs of the vaccine and tumor groups were significantly greater than those of the negative control group (*P*=0.05 and 0.005, respectively), indicating greater antibody titers in the vaccine and tumor groups than in the negative control group. 

***Effects of polyclonal antibody on HUVEC proliferation***

**Figure 4:**
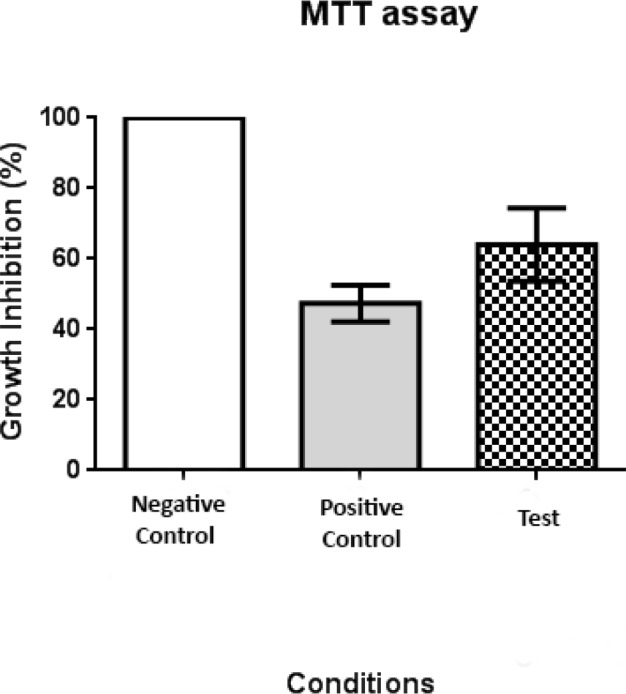
Human umbilical vein endothelial cell proliferation inhibition was determined by MTT assay

We investigated the effect of purified immunoglobulins from immunized mice sera on proliferation of VEGF-A-treated HUVEC by MTT assay. The negative control, positive control, and test cells all received 50 ng/ml of recombinant VEGF-A. The positive control cells also received 300 ng/ml of anti-VEGF monoclonal antibody, and the test cells received 80 μg/ml IgG from immunized mice. Proliferation was set at 100% for the negative control cells. Proliferation in the positive control and test cells was significantly lower than in the negative controls (Figure 4, *P*<0.001 for both groups).

***Inhibition of tumor growth in mice***

**Figure 5:**
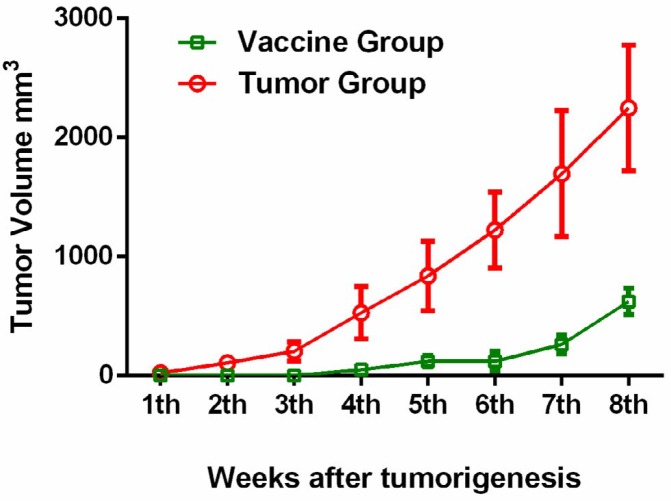
Tumor volumes in vascular endothelial growth factor (VEGF) peptide-vaccinated (vaccine group) and untreated (tumor group) mice 1-8 weeks after tumorigenesis with 4T1 cells

After injection of 4T1 cells, tumor size was determined in the vaccine and tumor group up to eight weeks. From the second week on, tumors were larger in the tumor group than in the vaccine group, and this difference was statistically significant at eight weeks (*P*<0.001). No tumor growth was observed in the vaccine group from weeks 1-6 (Figure 5). 

**Figure 6:**
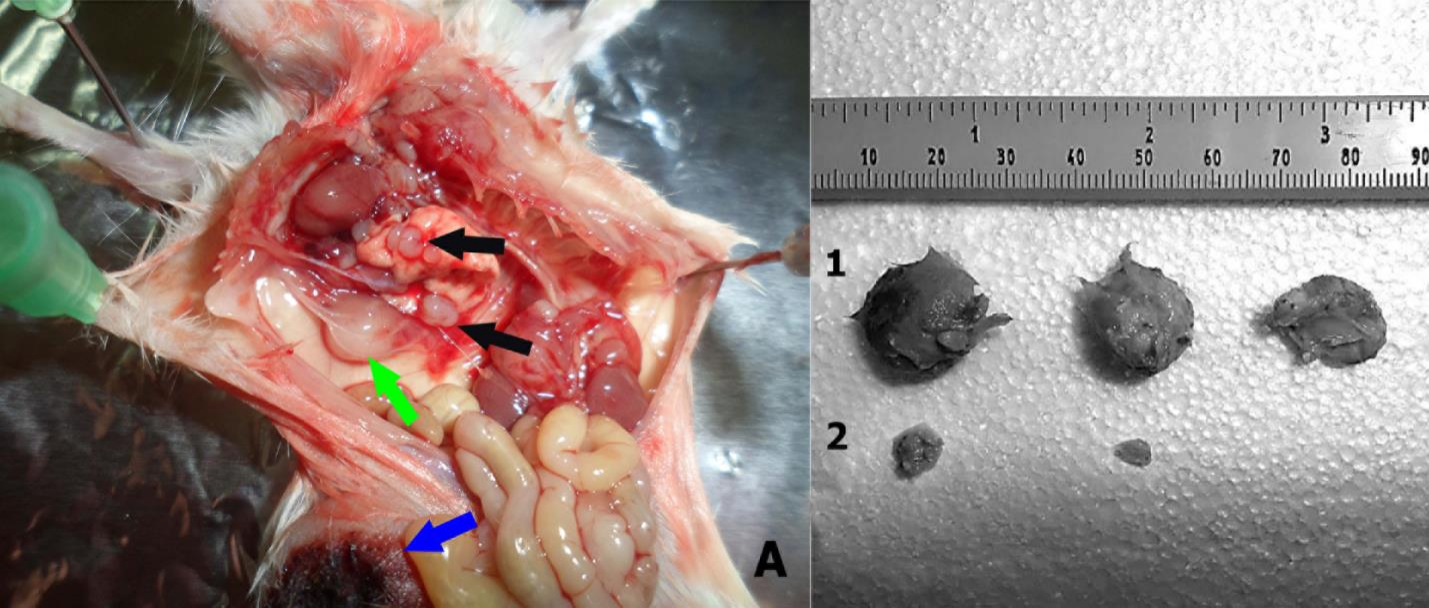
Tumor metastasis. (A) Primary tumor and metastases in lymph nodes and lung in one tumor group mouse inoculated with 4T1 tumor cells. Five weeks after injection of 5×10^5^ 4T1 tumor cells in mice, in addition to the primary tumor (blue arrow), metastases to lung (black arrows) and lymph node (green arrow) were observed. (B) Primary tumors size from tumor (1) and vaccine (2) group mice

Five weeks after inoculations with 4T1 cells, three mice from the vaccine and tumor groups were euthanized for tissues examination. Metastases were observed in the lung and liver tissues, primarily in the tumor group mice (Figure 6A and Table 1). In addition, primary tumors in the tumor group were significantly larger than those in the vaccine group (Figure 6B, *P*<0.05).

Lung and liver tissues from the vaccine group and tumor group mice were examined by H&E staining and light microscopy. Five weeks after inoculation with 4T1 cells, both lung and liver from the vaccine group mice appeared normal, while lung and liver tissues from the tumor group mice showed metastases, inflammation, necrosis, and infiltration of inflammatory cells. 

Following necropsy, all mice were examined for metastasis and any other pathological disorders. Fewer metastatic tumors and tumor-related pathologies were found in the lung and liver tissues of vaccine group than tumor group (Table 1). No metastasis was found in the brain tissue of either groups. 

***Survival rates of the studied groups***

**Figure 7:**
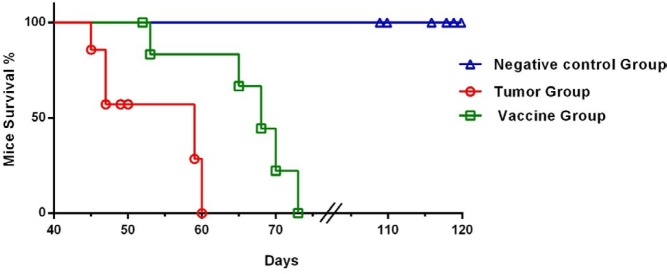
Survival rates of the negative control group, tumor group, and vaccine group. The tumor group and vaccine group mice were inoculated with 5×10^5^ 4T1 tumor cells. The negative control group mice received only phosphate-buffered saline (PBS) at the time of inoculation

The survival rates of the vaccine group mice were significantly greater than those of the tumor group mice (*P*<0.0001). All negative controls survived during the study (Figure 7). Notably, the vaccination steps did not show any side effects. So that, no significant changes were detected in behavior, feeding, body weight or appearance of the studied animals.

## Discussion

Because growth, expansion, and survival of tumor cells depend on angiogenesis, thus inhibition of angiogenic pathways could be a crucial strategy in cancer treatment (23). Active immunotherapy and vaccine design against VEGF and its receptors still remain topics of interest as therapeutic approaches for inhibition of angiogenesis (24, 25). Nowadays, various tumor-associated therapeutic proteins have been applied to elicit tumor-specific immune responses. One commonly used safe method relies on administration of recombinant peptides in presence or absence of adjuvants. Although these peptides have been shown to induce tumor-targeted immune responses, it seems that the anti-cancer potential of such approaches is limited (26). In 2001, Wei *et al.* applied the first VEGF-based cancer vaccine in the form of xenogenic DNA to evaluate its anti-tumoral effect on three different tumor models and observed that humoral immune response against VEGF could inhibit primary tumor growth (27). Kamstock *et al.* developed another xenogenic vaccine against VEGF and evaluated its efficacy in dogs with soft tissue sarcoma. In that study, DNA-liposome complexes were coupled with human VEGF165 and high antibody titers were achieved after immunization with the adjuvant-imbedded protein vaccine (28).

In another study, Rad *et al.* introduced the VEGF Kinoid vaccine based on human and murine VEGF isoforms. So that, a KLH-conjugated VEGF sequence was used for immunization, and after vaccination of the mice with Freund’s adjuvant, polyclonal anti-VEGF antibody was purified from the mice sera. Afterwards, they examined the inhibitory effect of polyclonal anti-VEGF on human colon carcinoma, as well as mouse and human rhabdosarcoma; consequently, they concluded that this approach achieved promising results for inhibition of metastasis (29). In another experiment, Gavilando *et al.* introduced CIGB-247 as a novel protein vaccine. They studied the immunogenicity of this protein in murine, rat, rabbit, and monkey models and reported no unfavorable hematological, biochemical, or histological side effects on the vital organs of the studied animals. This vaccine also did not show any side effects on normal behavior of the animals and demonstrated maintenance of desirable antibody titers after booster doses. This vaccine was a recombinant form of human VEGF, which was expressed in *Escherichia coli* and applied in parallel with small amounts of proteoliposome of the outer cell wall of *Neisseria meningitidis* as adjuvant. Immunization of mice with CIGB-247 significantly reduced tumor growth and increased animal survival rate and serum titer of anti-VEGF antibody (14-30). Afterwards, investigation of the safety and immunogenicity of CIGB-247 in human phase I clinical trial initiated and demonstrated some clinical benefits (31). 

Kaumaya *et al.* in 2010 developed a peptide vaccine consisting of synthetic peptides of VEGF as an antigen and T cell epitope of the measles virus fusion protein (MVF) protein as an adjuvant. After evaluation of efficiency of this peptide vaccine on inhibition of VEGFR2 signaling pathway (32), Wang *et al.* used these synthetic peptide vaccines in murine ovarian cancer model, and the development of high titers of antibody against synthetic peptides was in line with inhibition of angiogenesis in primary tumor models (33). 

In 2013, Kyutoku *et al.* designed DNA vaccine for neutralizing VEGF. For enhancing the immunogenicity of vaccine, hepatitis B virus core (HBc) antigen was considered as an epitope carrier. HBc-VEGF vaccine was evaluated in murine with colon carcinoma and showed humoral immune response that reduced formation of new vessels (34).

Unlike the above studies, the peptide vaccine in our study was designed based on immunogenic structures. Hence, as expected, and as the results of the immunoinformatics analysis showed, the selected peptide, in addition to sufficient antigenicity and ability to stimulate the body’s immune system had the least similarity to other proteins and probably might cause fewer side effects.

Similar to experiments of Rad *et al.* in 2007, kinoid technology against VEGF was used. KLH was conjugated to the designed peptide to stimulate the immune system and overcome tolerance to VEGF, as a self-antigen. The MTT assay revealed that purified IgG from the peptide-vaccinated mice inhibited VEGF-A-induced HUVEC proliferation, and this result was similar to the inhibitory effect of monoclonal anti-VEGF antibody. 

In this study, according to assessment in UniProt and other bioinformatics databases, we chose a 41-mer peptide. This 41-mer sequence was selected from a conserved part of VEGF molecule that did not differ in the amino acid sequence between the human-VEGF and mouse-VEGF. The selected 41-mer peptide stimulated a specific humoral immune response against VEGF. We demonstrated that *in silico*-designed peptide vaccines can inhibit metastasis *in vivo*. In addition to monoclonal antibodies, such as Bevacizumab that have adverse side effects and require repeated administrations, cancer vaccines offer promise for cancer patients. 

Our results demonstrated that vaccination with a VEGF peptide might produce an antibody response and inhibit primary tumor growth and metastasis.

Although anti-VEGF antibody was produced in the tumor group mice, tumor growth was significantly lower in the vaccine group mice (*P*<0.001), and no tumor growth was observed in this group until seven weeks after inoculation (Figure. 5). In some studies, decreases in tumor growth required multiple immunizations and results were not significant. Gavilando *et al.* showed decreased tumor growth using six immunizations and three different adjuvants over 36 days following inoculation of mice with cancer cells (11), and Wang *et al.* showed decreased tumor growth using four immunizations with a VEGF peptide vaccine conjugated to measles virus fusion protein over 45 days after inoculation of mice with cancer cells (28).

To evaluate metastasis, the use of appropriate cancer cell lines with effective invasion and metastatic properties is crucial. Because the immune system plays an important role in the development and progression of tumor, we used BALB/c mice and 4T1 breast cancer cells, which are highly tumorigenic and invasive, and unlike most tumor models, can spontaneously migrate from the primary tumor in the mammary gland to distal sites including lymph nodes, liver, lung, brain, and bone (35, 36). 

In this study, by using 4T1 cancer cells, we found less metastasis, necrosis, inflammation, vascularization, and micro-metastatic clones in livers and lungs of the vaccine group than in the tumor group mice (data not shown). Our findings indicate that the antibody response could significantly inhibit metastasis, and the mice showed no metastatic markers. Moreover, no brain metastases were found in the six mice, which were sacrificed five weeks after tumor growth. However, metastasis to liver and lung was obviously severe in the tumor group.

Previous studies showed that multiple immunization and application of adjuvants could decrease the rate of metastasis and increase survival rate, and it could be due to antibody titer rise. Our study was relatively short due to the rapid appearance of tumors and metastases following a single injection with 4T1 cells. In future studies, we will further examine the effects of multiple immunizations and adjuvants. In addition, the presence of a T cell epitope also allowed us to assess immunologic memory. The low survival rates of the mice in this study did not allow sufficient time to observe changes in antibody titers.

Application of an appropriate treatment and considering an optimal strategy for inhibition of tumor growth is a crucial item in cancer therapy. Recent studies have shown that combination therapies, especially combination of immunotherapy with chemotherapy, and usage of immune checkpoint inhibitors can provide hopeful results in clinic (37, 38). In this regard, designing and employing of peptide-based vaccines sounds a promising strategy (39). Therefore, considering the time limitation for hindering possibility of metastasis, it seems that anti-angiogenic vaccination strategies such as immunization against VEGF might be a promising method following initial diagnosis of cancer.

The major histocompatability complex (MHC) I and II were restricted to human leukocyte antigen A*0201 (HLA-A*0201) (Red) and HLA-DRB1* 1103/1104 (Highlight). The percentile rank cut off point was set at <5 (lower percentile ranks reflects good binding)

The negative control cells received 50 ng/ml of recombinant vascular endothelial growth factor (VEGF)-A only. The positive control cells received 50 ng/ml of recombinant VEGF-A plus 300 ng/ml of anti-VEGF monoclonal antibody. The test cells received 50 ng/ml of recombinant VEGF-A plus 80 μg/ml total IgG that was isolated from immunized mice. Proliferation for the negative control cells was set at 100%. Growth was significantly less in both the positive control and test cells than in the negative controls (both had *P<*0.001)

**Table 1:** Characterization of metastatic properties in lung and liver tissues. Vascularization, necrosis, inflammation, infiltration, and number of detectable metastases in lung and liver are shown

Mouse ID	Metastatic Tissues	Groups	Vascularization*	Necrosis**	Inflammation***	Infiltration %	No. of distinct histologically detectable metastasis	
31	Lung	Vaccine	0	0	0	0%		0
32			0	0	0	0%		0
33			+1	+1	Mo+	40%		3
21		Tumor Only	+1	0	Mo+	40%		10
22			+1	+3	Mo+	70%		4
23			+1	0	Mo+	60%		1
							
31	Liver	Vaccine	0	0	0	0%		0
32			0	0	0	0%		0
33			0	0	PMN+	40%		3
21		Tumor Only	+2	+2	PMN+	10%		11
22			+2	+1	0	5%		12
23			+1	0	0	0		1

*0 = absence of microscopic neoangiogenesis; 1= moderate neoangiogenesis

**0=no necrosis; =moderate; 2=severe

***0=no inflammatory mononuclear and polymorphonuclear cells; Mo+=inflammatory mononuclear cells; PMN+=inflammatory polymorphonuclear cells

Infiltration %=percent of inflammatory cell infiltration

## Conclusion

We showed that immunization with a VEGF peptide-conjugate can inhibit tumor growth and metastasis in BALB/c mice and might probably increase patient survival rate, too.
